# Contrast Induced Nephropathy: Efficacy of matched hydration and forced diuresis for prevention in patients with impaired renal function undergoing coronary procedures–CINEMA trial

**DOI:** 10.1016/j.ijcha.2022.100959

**Published:** 2022-02-01

**Authors:** Aram J. Mirza, Kashan Ali, Farhad Huwez, Abdulsalam Y. Taha, Farman J. Ahmed, Shahow A. Ezzaddin, Zana I. Abdulrahman, Chim C. Lang

**Affiliations:** a Department of Interventional Cardiology, Slemani Cardiac Hospital, Sulaymaniyah, Region of Kurdistan, Iraq; b Division of Molecular & Clinical Medicine, School of Medicine, Ninewells Hospital & Medical School, University of Dundee, UK; c Royal London Hospital, Hyper-acute Stroke Unit, Whitechapel, London, UK; d Department of Thoracic and Cardiovascular Surgery, College of Medicine, University of Sulaimani, Sulaymaniyah, Region of Kurdistan, Iraq; e Department of Family and Community Medicine, College of Medicine, University of Sulaimani, Sulaymaniyah, Region of Kurdistan, Iraq; f Shorsh General Hospital, Peshmarga Health Foundation, Ministry of Peshmarga, Region of Kurdistan, Iraq

**Keywords:** Contrast-induced nephropathy, Renal impairment, Contrast media, Coronary angiography, Percutaneous coronary intervention, Matched hydration and forced diuresis

## Abstract

**Background:**

Matched hydration and forced diuresis (MHFD) using the RenalGuard device has been shown to reduce contrast induced nephropathy (CIN) following coronary interventions.

**Aim:**

To evaluate the potential benefits of a non-automated MHFD protocol compared to current hydration protocol in prevention of CIN in patients with CKD.

**Methods:**

A total of 1,205 patients were randomized to either non-automated MHFD group (n = 799) or intravenous hydration control group (n = 406). The MHFD group received 250 ml IV normal saline over 30 min before the coronary procedure followed by 0.5 mg/kg IV furosemide. Hydration infusion rate was manually adjusted to replace the patient's urine output. When urine output rate reached > 300 ml/h, patients underwent coronary procedure. Matched fluid replacement was maintained during the procedure and for 4-hour post-treatment. CIN was defined conventionally as ≥ 25% or ≥ 0.5 mg/dl rise in serum creatinine over baseline.

**Results:**

CIN occurred in 121 of 1,205 (10.0%) patients in our study. With respect to the primary outcome, 64 (8.01%) of the MHFD patients developed CIN compared with 57 (14.04%) of the control group (p < 0.001).

**Conclusions:**

A non-automated MHFD protocol is an effective and safe method for the prevention of CIN in patients with CKD.

## 1 Introduction

Globally a growing number of patients receive coronary angiography and percutaneous coronary intervention, requiring contrast media. Although most contrast media are considered safe, at-risk individuals can develop contrast-induced nephropathy (CIN) which is defined as ≥ 25% increase in serum creatinine from the baseline value, or an absolute increase of at least 0.5 mg/dL (44.2 µmol/ L), 48–72 h after the administration of radiographic contrast media that is not attributable to other causes [1]. CIN is not an uncommon complication after coronary angiographic procedures with the reported incidence of 1–2% in the general population and as high as 50% in some high-risk patient subgroups [1], [2]. It is the third leading cause of hospital-acquired acute kidney injury (AKI) [3]. Moreover, the occurrence of CIN following cardiac catheterization procedures is associated with a significantly increased relative risk for serious adverse short- and long-term outcomes [4], [5], [6], [7], [8]. A recent study suggested ∼4-fold increased risk of 90-day death, need for dialysis, or persistent kidney impairment associated with CIN [9].

Although no known pharmaceutical treatment can effectively prevent or treat CIN, various preventative strategies including risk assessment before contrast media exposure, withdrawal of the nephrotoxic drugs, volume expansion with sodium chloride or sodium bicarbonate, hemofiltration or hemodialysis, and the optimal contrast media policy have been developed to CIN prevention. Peri-procedural intravascular hydration is the single most important measure comprehensively proven to prevent the occurrence of CIN, recommended by guidelines [10], [11], [12], [13]. There are several specific hydration strategies, but an optimal strategy has not been established yet [14], [15], [16]. Several small randomized controlled trials (RCTs) and prospective studies have shown that in patients with chronic kidney disease (CKD), furosemide-induced forced diuresis with matched hydration using the RenalGuard system can prevent the occurrence of CIN [17], [18], [19], [20]. Matched hydration is a term used to describe the technique of delivering intravenous fluid matched to urine output. The RenalGuard system was developed in order to achieve precise, real-time automated fluid matching [21]. A recent *meta*-analysis of high-volume forced diuresis with matched hydration using the RenalGuard medical device system revealed a significant reduction in the risk of CIN, major adverse cardiac event rate, and the need for renal replacement therapy [22]. However, the RenalGuard device is not widely available and affordable especially in low-middle income countries. It is not known if a non-automated matched hydration with forced diuresis protocol would also have beneficial effects in preventing CIN in patients identified to have CKD. The purpose of our study was to evaluate the potential benefits of a non-automated matched hydration and forced diuresis (MHFD) protocol in the prevention of CIN in patients identified to have CKD undergoing coronary procedures compared to current hydration protocol.

## 2 Methods

**Study design:** This was a prospective open-label randomized controlled trial (ISRCTN Registry Number 72194653) conducted in a pragmatic manner in our hospital over 28 months commencing in November 2017. All patients provided written informed consent for participation in this trial that was approved by the Ethical Committee of our Medical College and conducted according to the principles of the Declaration of Helsinki and Good Clinical Practice.

**Fig. 1:**
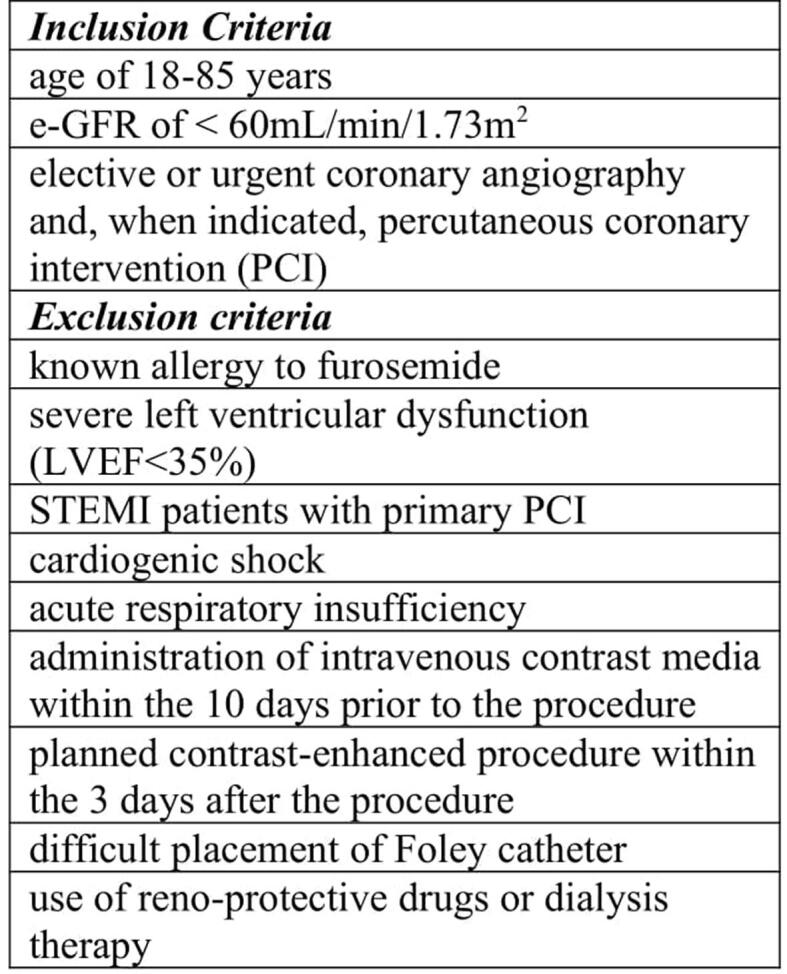
Inclusion and exclusion criteria for the study population.

**Study Population:** We enrolled 1,205 consecutive patients with CKD scheduled for coronary angiography at our hospital. Inclusion criteria were age of 18–85 years, estimated glomerular filtration rate (e-GFR) of < 60 ml/min/1.73 m^2^ and elective or urgent coronary angiography and, when indicated, percutaneous coronary intervention (PCI). Exclusion criteria were known allergy to furosemide, severe left ventricular dysfunction, primary or rescue PCI, cardiogenic shock, acute respiratory insufficiency, administration of intravenous contrast media within the 10 days prior to the procedure, planned contrast-enhanced procedure within the 3 days after the procedure, difficult placement of Foley catheter, use of reno-protective drugs or dialysis therapy. (Fig. 1)

In our study, CKD was defined as an e-GFR of < 60 ml/min/1.73 m^2^ based on the recommendations of the National Kidney Foundation [23]. Patients were admitted to the hospital one day before the procedure for assessment of e-GFR using the Modification of Diet in Renal Disease (MDRD) formula [24]. Significant left ventricular systolic dysfunction was defined as prior echocardiogram derived Left Ventricular Ejection Fraction (LVEF) < 40 %.

**Study Protocol:** Patients were randomized into 2 groups in a 2:1 ratio to the study group (matched hydration and forced diuresis; MHFD) and the control group (intravenous hydration) respectively.

In the MHFD group, a peripheral intravenous cannula (18-gauge) was inserted into a peripheral vein of the arm. A Foley catheter was also positioned in the urinary bladder for urine collection. The cannula was connected with a bag of normal saline for fluid infusion. To avoid fluid over-load and hypovolemia, the amount of normal saline delivered to the patient was matched with the volume of urine produced by the patient.

Before the coronary procedure, MHFD treatment was commenced with an initial intravenous bolus (250 ml) of normal saline solution over 30 min followed by a bolus dose of furosemide (0.5 mg/kg). Injection of contrast media was deferred until urine flow rate exceeded 300 ml/hour. Matched Hydration was continued throughout the cardiac catheterization procedure and up to 4 h after the procedure. Matched Hydration was achieved by initially giving normal saline intravenously at a rate of 200 ml/h, followed by adjustment in the infusion rate to match the urine output after every 5 min. The latter was recorded by nursing staff on the bedside flow sheet. Urine flow rate was maintained at > 300 ml/hour with additional doses of furosemide if necessary.

In the control group, patients received a continuous intravenous infusion of isotonic saline at a rate of 1–1.5 ml/kg for at least 12 h before and 12 h after the procedure.

The Foley catheter was removed 12 h after the procedure. Peri-procedural medical therapy, PCI technique, and contrast dose were left to the preference of the interventional cardiologist in charge of the patient. Serum creatinine was evaluated at baseline, the day of coronary angiography, 48 h after the procedure, and at hospital discharge.

**Study Endpoints:** The primary endpoint was the development of CIN, defined as a ≥ 25% or ≥ 0.5 mg/dl rise in serum creatinine over baseline at 48 h post-procedure. Secondary endpoints were CIN requiring renal replacement therapy, significant arrhythmias, cardiogenic shock, pulmonary edema and death.

**Statistical Analysis:** Power calculations was based on the MYTHOS Trial that reported the development of CIN in 4.6% of the study group and 18% in the control group [18]. With an enrollment ratio of 2:1, a significance level of 0.05 and 80% power, 69 subjects in control group and 138 subjects in study group were required to demonstrate the expected difference in the incidence of CIN between groups.

Statistical analysis was performed with SPSS, version 21. Continuous variables were expressed as mean ± standard deviation (SD) or median (interquartile range) if they followed a normal or non-normal distribution, respectively. Categorical variables were reported as number and percentages in brackets. The distribution of continuous variables was assessed using Kolmogorov-Smirnov test. Chi-square tests were used to compare the categorical data between the two groups while continuous variables among the two groups of the study were compared with independent sample *t*-test. As some of the data were not normally distributed, the mean rank was useful for comparing the central tendency (group center) of compared groups. The difference in the mean rank between the 2 groups was assessed by non-parametric test (Mann-Whitney). A *p* value of 0.05 was required for statistical significance.

## 3 Results

A total of 1,205 consecutive patients (mean age: 62.3 ± 7.5 years, 698 men) with CKD (mean e-GFR: 46.37 ± 10.25 ml/min/1.73 m^2^) were included in the study. Of them, 1,086 underwent elective procedures and 119 underwent urgent angiography because of a non-ST elevation myocardial infarction (NSTEMI). The treatment assignment between the 2 groups was determined by randomization in a 2:1 ratio with 799 patients randomized to the MHFD group and 406 to the control group. No patients were lost to follow-up and all eligible patients were analyzed.

Baseline characteristics of the 2 groups are given in Table 1. The MHFD group patients were younger compared to the control group (Age 62.3 ± 7.5 vs. 65.5 ± 8.2 years, p < 0.001). Diabetes (DM), hypertension (HTN), smoking and peripheral arterial disease (PAD) were more prevalent in the study group than the control group. There were more patients requiring insulin in the MHFD group (p < 0.0001)

**Table 1:** Baseline Characteristics of the Study Patients.

	Study group (MHFD) n = 799	Control group (IVH) n = 406	P value
**Baseline clinical characteristics**			
Age	62.3 ± 7.5	65.5 ± 8.2	< 0.001
Men	457 (57.2%)	241 (59.4%)	0.47
Diabetes Mellitus	451 (56.4%)	192 (47.3%)	0.003
Hypertension	587 (73.5%)	234 (57.6%)	< 0.001
Smokers	320 (40.1%)	114 (28.1%)	< 0.001
PAD	353 (44.2%)	103 (25.4%)	< 0.001
LVEF%	51.17 ± 9.53	52.02 ± 9.94	0.15
**No. of procedures performed**			
Elective procedures	717 (89.7%)	369 (90.9%)	0.53
Urgent procedures	82 (10.3%)	37 (9.1%)	
**Procedure**			
Coronary angiography	102 (12.8%)	70 (17.2%)	0.04
PCI	694 (86.9%)	327 (80.5%)	0.004
PCI- CTO	3 (0.4%)	9 (2.2%)	0.002
Contrast volume (ml)	152.82 ± 66.16	146.19 ± 67.99	0.10
**Lab measures**			
e - GFR	46.37 ± 10.25	46.29 ± 9.6	0.90
Creatinine (mg / dl)	1.52 ± 0.25	1.51 ± 0.20	0.51
Creatinine 48 h after the procedure (mg / dl)	1.75 ± 0.33	1.69 ± 0.32	0.004
HbA1c	6.85 ± 1.27	6.99 ± 2.93	0.23
S Cholesterol (mg/dl)	152.69 ± 53.79	163.08 ± 33.71	< 0.001
S LDL (mg/dl)	94.12 ± 36.11	107.67 ± 49.18	< 0.001
S HDL (mg/dl)	40.26 ± 6.35	40.18 ± 5.23	0.84
TG (mg/dl)	176.42 ± 92.17	205.41 ± 80.74	< 0.001
Hb%	13.19 ± 1.30	13.49 ± 1.28	< 0.001
WBC	9.12 ± 2.06	9.17 ± 1.98	0.68
Lymphocyte	3.53 ± 0.85	3.48 ± 0.88	0.32
Neutrophil	5.35 ± 1.07	5.30 ± 0.98	0.44
Platelets	354.58 ± 127.47	349.43 ± 128.72	0.51
**Medications**			
Diuretics	277 (34.7%)	128 (31.5%)	0.28
Insulin	242 (30.3%)	85 (20.9%)	< 0.001
Oral hypoglycemic agents	200 (25.1%)	102 (25.1%)	0.97
ACE inhibitors	269 (33.7 %)	124 (30.5%)	0.27
MRAs	70(8.8%)	35 (8.6%)	0.94
ARBs	350 (43.8%)	122 (30%)	<0.001
Beta blockers	399 (49.9%)	183 (45.1%)	0.11
CCB	182 (22.8%)	96 (23.6%)	0.36
Ranolazine	84 (10.5%)	62 (15.3%)	0.02
Antiplatelets	794 (99.4%)	404 (99.5%)	0.77
Nitrates	209 (26.2%)	97 (23.9)	0.39
Statins	639 (80.0%)	342 (84.2%)	0.07

ACE, Angiotensin Converting Enzyme; ARB, Angiotensin Receptor Antagonist; CCB, Calcium Channel Blocker; CTO, Chronic Total Occlusion; e-GFR, estimated Glomerular Filtration Rate; Hb, Haemoglobin; HbA1c, Glycosylated Haemoglobin; HDL, High Density Cholesterol; LDL, Low Density Cholesterol; LVEF, Left Ventricular Ejection Fraction; MRA, Mineralocorticoid Receptor Antagonist; PAD, Peripheral Arterial Disease; PCI, Percutaneous Coronary Intervention; TG, Triglycerides; WBC, White Blood Cells.

Both groups were comparable in regard to the rate of elective and urgent procedures: 717 (89.7%) vs. 369 (90.9%) and 82 (10.3%) vs. 37 (9.1%) respectively. PCI were more frequently performed in the MHFD group compared to the control group: 694 (86.9%) vs. 327 (80.5%) (p = 0.004).

The frequency of additional CIN risk factors including baseline eGFR and contrast volume and use of nephrotoxic drugs were similar between the two groups.

The target urine flow (>300 ml/hour) was reached in all patients in the MHFD group.

**Fig. 2:**
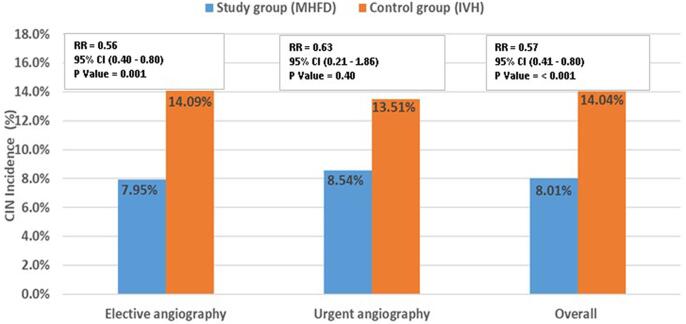
Incidence of CIN in All Study Patients and in Those Undergoing Elective or Urgent Coronary Angiography.

Overall CIN occurred in 121 of 1,205 (10.0%) patients in our study. CIN was significantly higher among patients who underwent elective procedures (n = 109, 9.0%) than those who underwent urgent procedures (n = 12, 1.0%) (p < 0.05). With respect to the primary outcome, 64 (8.01%) of the MHFD patients developed CIN compared with 57 (14.04%) of the control group (p < 0.001). When we compared the development of CIN in elective procedures compared to emergency procedures, the MHFD treatment was particularly effective in patients undergoing elective procedures as compared to emergency procedures. (Fig. 2)

No significant MHFD-associated complications were observed. (Table 2) In-hospital renal failure requiring dialysis occurred in 4 patients in the control group (1.0%) compared with 9 patients (1.1%) in the MHFD group (*p = NS*).

**Table 2:** Post-Procedural Complications.

	Study Group (MHFD)	Control Group	p value
CIN requiring RRT	9 (1.1%)	4 (1.0%)	p = NS
Significant arrhythmia	1 (0.1%)	0 (0%)	p = NS
Cardiogenic shock	0 (0%)	0 (0%)	p = NS
Pulmonary edema	3 (0.4%)	3 (0.7%)	p = NS
Death	0 (0%)	0 (0%)	p = NS
All clinical events	13 (1.6%)	7 (1.7%)	p = NS

CIN, Contrast Induced Nephropathy; RRT, Renal Replacement Therapy.

## 4 Discussion

Prevention of CIN is of paramount importance, with an ever-increasing number of coronary angiographies and interventions being done. Current clinical practice guidelines recommended adequate hydration, minimizing volume of contrast media and using *iso*-osmolar or low-osmolar contrast agents [10], [11], [12], [13], [25]. The role of nephroprotective drugs in preventing CIN is not recommended.

Adequate hydration remains the mainstay of CIN prevention since early 1970s [26]. This was due to observation that dehydration would exacerbate renal insufficiency in a patient exposed to contrast media. Hydration increases the intravascular blood volume, suppresses the renin-angiotensin-aldosterone system, and promotes dilution and rapid evacuation of contrast media. Contrast Media Safety Committee recommends an intravenous regime of 1.0–1.5 ml/kg/hour for at least 6 h before and after contrast media administration, [13] though concerns regarding volume overload for patients undergoing cardiac catheterization procedures often lead to insufficient pre-hydration. Furosemide may decrease the nephrotoxic effect of contrast agents, first by increasing the urine flow and hence diluting the contrast media, and second by blocking tubular sodium reabsorption in the loop of Henle, thus decreasing tubular workload and associated oxygen requirement. However, the use of furosemide alone is controversial since it decreases the effective circulating volume and prostaglandin mediated vasodilation with the potential dehydration. This concern has prompted the exploration of the use of diuretics together with hydration. Several studies have evaluated hydration and diuresis with mixed results [27], [28]. However in these older studies there was a lack of adequate matching between hydration and urine flow. The PRINCE trial reported a reduction in CIN with forced diuresis resulting in a mean urine flow rate of above 150 ml/min [29]. However, in this trial, <30% of the enrolled patients reached that urine flow target, even when a forced diuresis approach was used (furosemide plus dopamine plus mannitol administration). Also, fluid administration matched to urine output was commenced after started the cardiac catheterization procedure. This contrasts with our study that required matched Hydration to be continued throughout the catheterization procedure and up to 4 h after the procedure, and nearly all patients reached the urine flow target of > 300 ml/hour. On the other hand, Majumdar et al. [30] reported a higher (50%) CIN rate in forced diuresis patients than in those who received saline infusion only. However, there are several differences between the study by Majmudar et al and our study. Unlike our study, the study by Majmudar et al was a small study which used a different treatment protocol including hypotonic saline solution and continuous infusion of furosemide. Also, they had included a greater proportion of patients with more severe CKD (average eGFR: 27 ml/min/1.73 m^2^).

The RenalGuard™ System (PLC Medical Systems, Inc. Franklin, MA, USA) is a device that can guide the physician in achieving high urine output with a low furosemide dose while simultaneously balancing urine output and venous fluid infusion to minimize the risk of overhydration or underhydration. Several studies have demonstrated that the approach of controlled, forced diuresis using the RenalGuard therapy is more effective than the conventional therapy in preventing CI-AKI in high-risk patients [17], [18], [19], [20]. However, the automated RenalGuard system is not widely available especially in many low- and middle-income countries. In our study, we have shown in randomized controlled trial the value of a non-automated matched hydration and forced diuresis in the prevention of CIN in patients with CKD who had undergone coronary procedures. The main finding of this study is the statistically significant lower rate of CIN among patients who received non-automated matched hydration and forced diuresis compared to the control group (8.01% vs. 14.04%, *p* < 0.001). In subgroups analysis, the incidence of CIN was not statistically different in patients undergoing urgent coronary procedures. This is likely due to the small sample size of patients who underwent urgent procedures in our study sample.

It is also noteworthy that baseline mean serum creatinine was similar in both MHFD and control groups. Although the MHFD group had younger patients and had low number of CTO-PCIs performed, overall MHFD group had higher rate of atherosclerosis-related risk factors such as DM, HTN, smoking and PAD, suggesting a higher risk of CIN in this group. Despite this increased risk profile, the rate of CIN was lower in the MHFD group. The magnitude of benefit seen in our study is similar to that achieved by MYTHOS trial that examined the benefits of furosemide-induced high-volume diuresis and maintenance of intravascular volume through automatic RenalGuard matched hydration system for the prevention of CIN in high-risk patients undergoing coronary procedures [18]. Our study suggests that a non-automated MHFD method can be used as an effective alternative to the RenalGuard system.

## 5 Study limitations

This study has several limitations. First, this was a single-center open-label study. However, this study is conceived a pragmatic study with limited funding and therefore it was not possible to blind the study. Secondly, patients with left ventricular systolic dysfunction were not included. Thirdly the results from this trial should not be applied to patients undergoing primary PCI and especially to those with severe impairment of renal function, as the mean eGFR was 46.37 ± 10.25 ml/min/1.73 m2. It should however be noted that to the best of our knowledge, this is the largest study of non-automated matched hydration forced diuresis in patients with CKD undergoing coronary interventions.

## 6 Conclusion

Similar to previously published studies, in the CINEMA study, we showed that furosemide-induced diuresis with maintenance of intravascular volume through a non-automated matched hydration is effective and safe method in reducing the risk of CIN in CKD patients undergoing coronary procedures.

## Declaration of Competing Interest

The authors declare that they have no known competing financial interests or personal relationships that could have appeared to influence the work reported in this paper.
